# Supplementary material for: Application of Acoustic Cardiography in Assessment of Cardiac Function in Horses with Atrial Fibrillation Before and After Cardioversion
Source: Animals (Basel). 2025 Jul 7;15(13):1993. doi: 10.3390/ani15131993 (PMC12248963; doi:10.3390/ani15131993)

Figure S1: Illustration of Audicor®.

Electrocardiogram and phonocardiogram by the Audicor® Dx Patch device. The vertical lines shoe the timing of the measurements (onset of QRS complex, S1, S2). On the two-dimensional representation of the phonocardiogram (a and b) the sound frequency is shown on the y-axis and the acoustic energy is displayed using a color code (low energy signals in blue, intermediate energy signals in yellow, high energy signals in red). In the three-dimensional illustrations (c and d) sound frequency is shown on the z-axis, acoustic energy on the y-axis and time on the x-axis. The fiducial points for S1, S2 and S4 are placed at the respective peak amplitudes of the heart sounds, as identified by the algorithm. Amplitude standardization and signal filtering are based on proprietary processing algorithms developed for human data. The figure serves as an illustration of signal alignment and the derivation of systolic time intervals, rather than for quantitative evaluation.

a) Electrocardiogram and two-dimensional phonocardiogram by the Audicor® Dx Patch device displaying atrial fibrillation. b) Electrocardiogram and two-dimensional phonocardiogram by the Audicor® Dx Patch device of horse from (a) 1d after conversion to normal sinus rhythm. c) Three-dimensional illustration in low temporal solution of the phonocardiogram shown in (a), showing the two heart sounds S1 and S2. d) Three-dimensional illustration I low-temporal resolution of the phonocardiogram shown in (b) showing three heart sounds S1, S2 and S4. EMAT, electromechanical activation time; LVST, left ventricular systolic time; S1, first heart sound; S2, second heart sound; S4, fourth heart sound.

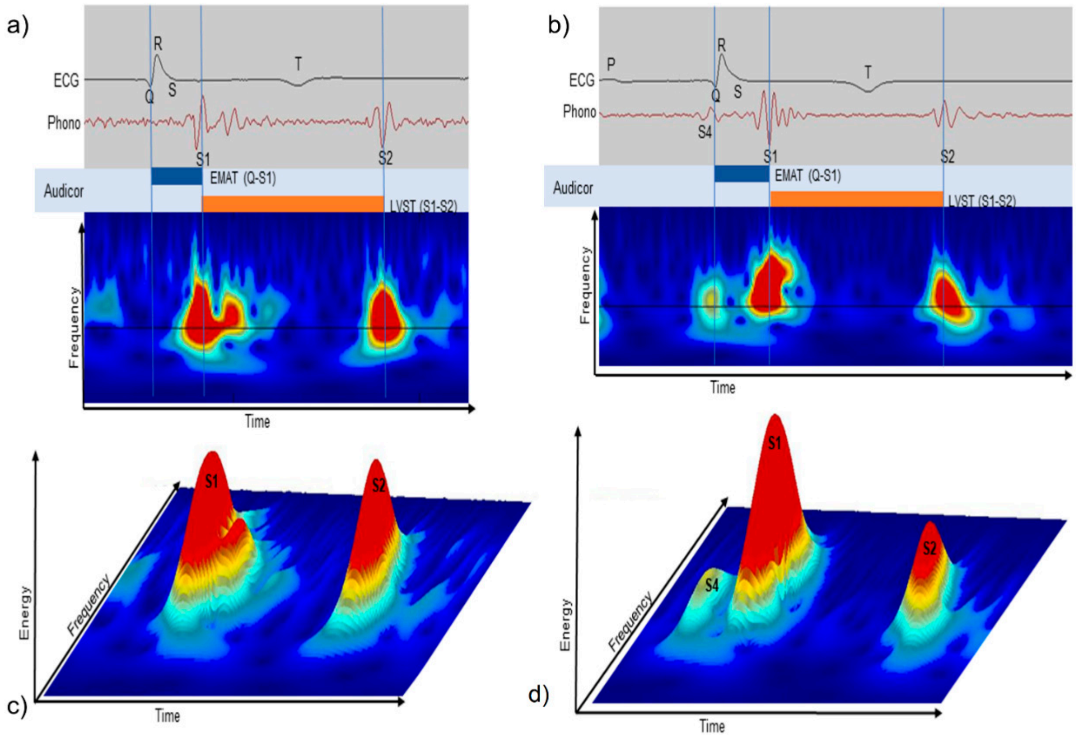

Supplement: Supplementary file 1 [file animals-15-01993-s001.zip › Figure S1_Illustration Audicor.pdf]
