# Supplementary material for: Application of Acoustic Cardiography in Assessment of Cardiac Function in Horses with Atrial Fibrillation Before and After Cardioversion
Source: Animals (Basel). 2025 Jul 7;15(13):1993. doi: 10.3390/ani15131993 (PMC12248963; doi:10.3390/ani15131993)
Supplement: Supplementary file 1 [file animals-15-01993-s001.zip › Table S1_Number of cycles analyzed per timepoint.pdf]

Table S1: Number of echocardiographic variables analyzed based on fewer than three cardiac cycles

Measurements derived from fewer than three cardiac cycles resulted primarily from technical limitations during image acquisition, including shorter recording durations, suboptimal image quality, and for color tissue Doppler Imaging (cTDI), suboptimal Doppler angle alignment relative to myocardial motion.

Variable	Unit	AF day -1	NSR day 1	NSR day ≥2
2DE, right-parasternal long-axis view of the left atrium and left ventricle, optimized to image the LA				
LADmax (500)	cm	1/22	2/21	0/17
LAAmax (500)	cm^2	1/22	2/21	0/17
active LA FAC	%	n/a	2/15	0/15
LA RI	%	1/22	2/21	0/17
active:total LA AC	-	n/a	2/14	0/14
2DE, left-parasternal long-axis view of the left atrium and left ventricle, optimized to image the LA				
LADllx-max (500)	cm	2/21	0/22	0/17
2DE, right-parasternal short-axis view of the aorta and the left atrium, optimized to image the LA and the LA appendage				
LAsxAmax (500)	cm^2	1/20	3/21	1/17
Linear measurements of LV size and function: Anatomic M-mode, right-parasternal short-axis view at the chordal level				
LVIDd (500)	cm	8/20	6/21	6/16
LADmax/LVIDd	-	8/20	6/21	6/16
RWTd	-	8/20	6/21	6/16
LV FS	%	8/20	6/21	6/16
Volumetric estimates of LV size and function using single-plane Simpson's method of disks: 2DE, right-parasternal long-axis four chamber view, optimized to image the LV				
LVIVd (500)	mL	1/19	0/21	2/17
LV EF	%	1/19	0/21	2/17
SV	mL	1/19	0/21	2/17
CO	L/min	1/19	0/21	2/17
Pulsed-wave tissue Doppler imaging, right-parasternal short-axis view at the chordal level, cursor placed on LV free wall				
Em	cm/s	3/15	2/16	2/12
Am	cm/s	n/a	2/10	2/10

Em/Am	-	n/a	2/10	2/10
PEPm	msec	3/15	2/16	2/12
ETm	msec	3/15	2/16	2/12
PEPm/ETm	-	3/15	2/16	2/12
IMPm	-	3/15	2/16	2/12
Sm	cm/s	3/15	2/16	2/12
Color tissue Doppler imaging, right-parasternal short-axis view at the chordal level, cursor placed on LV free wall				
Em	cm/s	18/20	16/21	10/16
Am	cm/s	n/a	11/13	10/13
Em/Am	-	n/a	11/13	10/13
PEPm	msec	18/20	16/21	10/16
ETm	msec	18/20	16/21	10/16
PEPm/ETm	-	18/20	16/21	10/16
IMPm	-	18/20	16/21	10/16
Sm	cm/s	18/20	16/21	10/16

For detailed explanation of variables see Table S2. LADmax and LADllx-max, maximum left atrial diameters; LAAmax and LAsxAmax, maximum left atrial areas; active LA FAC, left atrial active fractional area change; LA RI, left atrial reservoir index; active:total LA AC, ratio of active-to-total left atrial area change; LVIDd, left ventricular diameter at end-diastole; LVIVd, left ventricular volume at end-diastole; RWTd, relative LV wall thickness at end-diastole; LV FS, left ventricular fractional shortening; LV EF, left ventricular ejection fraction; SV, stroke volume; CO, cardiac output; Am, late-diastolic LV wall motion velocity at the time of atrial contraction; Em, early-diastolic LV wall motion velocity during the phase of rapid ventricular filling; Em/Am, ratio of Em-to-Am; PEPm, pre-ejection period; ETm, ejection time; PEPm/ETm, ratio of PEPm-to-ETm; IMPm, index of myocardial performance; Sm, wall motion velocity during LV ejection.
