# Supplementary material for: Application of Acoustic Cardiography in Assessment of Cardiac Function in Horses with Atrial Fibrillation Before and After Cardioversion
Source: Animals (Basel). 2025 Jul 7;15(13):1993. doi: 10.3390/ani15131993 (PMC12248963; doi:10.3390/ani15131993)
Supplement: Supplementary file 1 [file animals-15-01993-s001.zip › Table S2_List of echocardiographic variables.pdf]

Table S2: List of the echocardiographic variables measured for the purpose of this study (see [9, 20-22, 41, 42]).

**Left atrium (LA)**		
2DE, right-parasternal long-axis view of the left atrium and left ventricle, optimized to image the LA		
LADmax	cm	Internal left atrial diameter measured at the widest distance parallel to the mitral valve annulus during maximum atrial filling (at end-systole, one frame before mitral valve opening)
LADmax (500)	cm	LADmax corrected to a BWT of 500kg
LAAmax	cm^2	Internal left atrial area measured during maximum atrial filling (at end-systole, one frame before mitral valve opening)
LAAmax (500)	cm^2	LAAmax corrected to a BWT of 500kg
LAAa	cm^2	Internal left atrial area measured at the time immediately before start of atrial contraction (at onset of the electrocardiographic P wave)
LAAmin	cm^2	Internal left atrial area measured at the end of atrial contraction (at end-diastole, at the time of mitral valve closure)
2DE, left-parasternal long-axis view of the left atrium and left ventricle, optimized to image the LA		
LADllx-max	cm	Left atrial diameter measured at the widest distance during maximum atrial filling (at end-systole, one frame before mitral valve opening)
LADllx-max (500)	cm	LADllx-max corrected to a BWT of 500kg
2DE, right-parasternal short-axis view of the aorta and the left atrium, optimized to image the LA and the LA appendage		
LAsxAmax	cm^2	Internal area of the left atrium during maximum atrial filling (at time of aortic valve closure)
LAsxAmax (500)	cm^2	LAsxAmax corrected to a BWT of 500kg
Calculated variables of LA mechanical function		
active LA FAC	%	Active fractional area change of the LA [active LA FAC = (LAAa – LAAmin) / LAAa × 100]
LA RI	%	LA reservoir index [LA RI = (LAAmax – LAAmin) / LAAmin × 100]
active:total LA AC	-	Ratio of active-to-total LA area change [active:total LA AC= (LAAa – LAAmin) / (LAAmax – LAAmin)]
**Left ventricle (LV)**		
Linear measurements of LV size and function: Anatomic M-mode, right-parasternal short-axis view at the chordal level		
LVIDd	cm	Left ventricular diameter at end-diastole
LVIDd (500)	cm	LVIDd corrected to a BWT of 500kg
LADmax/LVIDd	-	LADmax-to-LVIDd ratio
RWTd	-	Relative wall thickness at end-diastole [RWTd = (LVFWd + IVSd) / LVIDd]
LV FS	%	Left ventricular fractional shortening [LV FS = (LVIDd – LVIDs) / LVIDd × 100]

Volumetric estimates of LV size and function using single-plane Simpson's method of disks: 2DE, right-parasternal long-axis four chamber view, optimized to image the LV		
LVIVd	mL	Left ventricular volume at end-diastole
LVIVd (500)	mL	LVIVd corrected to a BWT of 500kg
LV EF	%	Left ventricular ejection fraction [LV EF = (LVIVd – LVIVs) / LVIVd × 100]
SV	mL	Stroke volume [SV = (LVIVd – LVIVs)]
CO	L/min	Cardiac output [CO = SV x heart rate]
Pulsed-wave and color tissue Doppler imaging, right-parasternal short-axis view at the chordal level, cursor placed on LV free wall		
Em	cm/s	Early-diastolic peak radial LV wall motion velocity
Am	cm/s	Late-diastolic peak radial LV wall motion velocity
Em/Am	-	Em-to-Am ratio
PEPm	msec	Pre-ejection period
PEPm-c	%	Rate-corrected PEPm = PEPm expressed as % of the length of the corresponding cardiac cycle (electrocardiographic RR interval) [PEPm-c (%) = PEPm / RR x 100]
ETm	msec	Ejection Time
ETm-c	%	Rate-corrected ETm = ETm expressed as % of the length of the corresponding cardiac cycle (electrocardiographic RR interval) [ETm-c (%) = ETm / RR x 100]
PEPm/ETm	-	PEPm-to-ETm ratio
IMPm	-	Index of myocardial performance
Sm	cm/s	Ejection velocity
