# Supplementary material for: Application of Acoustic Cardiography in Assessment of Cardiac Function in Horses with Atrial Fibrillation Before and After Cardioversion
Source: Animals (Basel). 2025 Jul 7;15(13):1993. doi: 10.3390/ani15131993 (PMC12248963; doi:10.3390/ani15131993)
Supplement: Supplementary file 1 [file animals-15-01993-s001.zip › Table S3_List of Audicor_acoustic cardiography variables.pdf]

Table S3: List of Audicor® acoustic cardiography variables.

Variable	Unit	Definition
HR	min^{-1}	Heart rate
QRS	msec	QRS duration
QTc	msec	Rate-corrected QT interval (Bazett's equation)<br>QTc = QT/√RR, where QT = QT interval and RR = preceding RR interval
EMAT	msec	Electromechanical activation time = Time interval between onset of the electrocardiographic Q wave and the first heart sound (S1)
LVST	msec	Left ventricular systolic time = Time interval between the first (S1) and the second heart sound (S2)
EMATc	%	Rate-corrected EMAT = EMAT expressed as % of the length of the corresponding cardiac cycle (electrocardiographic RR interval)
LVSTc	%	Rate-corrected LVST = LVST expressed as % of the length of the corresponding cardiac cycle (electrocardiographic RR interval)
S3	-	Power (= function of the intensity and persistence) of the third heart (S3) sound, expressed as an index ranging between 0 and 10
S4	-	Power (= function of the intensity and persistence) of the fourth heart (S4) sound, expressed as an index ranging between 0 and 10
SDI	-	Systolic Dysfunction Index = Function of QRS duration, QT interval, EMATc and intensity of S3, expressed as an index ranging between 0 and 10.<br>SDI = f([QRS] × [QT] × [S3] × [EMATc])
EMATc (≥15%)	%	Per cent detected 10-second segments in which EMATc is equal to or exceeding the threshold value of 15%
S3 (≥5)	%	Per cent detected 10-second segments in which S3 is equal to or exceeding the threshold value of 5
S4 (≥5)	%	Per cent detected 10-second segments in which S4 is equal to or exceeding the threshold value of 5
SDI (≥5)	%	Per cent detected 10-second segments in which SDI is equal to or exceeding the threshold value of 5
SDI (≥7.5)	%	Per cent detected 10-second segments in which SDI is equal to or exceeding the threshold value of 7.5
