# Supplementary material for: Application of Acoustic Cardiography in Assessment of Cardiac Function in Horses with Atrial Fibrillation Before and After Cardioversion
Source: Animals (Basel). 2025 Jul 7;15(13):1993. doi: 10.3390/ani15131993 (PMC12248963; doi:10.3390/ani15131993)
Supplement: Supplementary file 1 [file animals-15-01993-s001.zip › Table S4_Reproducibility of snapshots variables.pdf]

Table S4: Reproducibility of "snapshot" variables.

A pilot study was performed to determine the reproducibility of the Audicor® variables when using the median out of 5 consecutive snapshots compared to a single snapshot. In a previously used subset of repeated recordings from 10 randomly selected healthy horses [40] five consecutive 10-s snapshots were analyzed to assess the reproducibility of the variables. Test reproducibility was quantified based on the three repeated measurements obtained in each of the 10 randomly selected horses. Within-subject variance for repeated measurements (residual mean square), determined by a one-way ANOVA with horses as groups, was used for the quantification of reproducibility. The within-subject standard deviation (sw) was calculated as the square root of the residual mean square. Measurement variability was reported as coefficient of variation (CV) and as repeatability coefficient (RC) according to the British Standard Institution (BSI). The CV as a per cent value was calculated as CV = sw/mean x 100. The RC is the absolute value below which the difference between two measurements will lie with 95 % probability and is calculated as 1.96 x √2 x sw = 2.77 x sw [46]. The RC is clinically more applicable and standardized compared to the CV and it considers uncertainty of the point estimates and uncertainty of prediction associated with repeated measurements [47]. By comparing the RC to the magnitude of change observed in a variable over time, one can assess if the change is a result of measurement error and physiologic variability (observed change ≤RC) or a true change over time (observed change >RC) [40]. As the average out of 5 consecutive snapshots was more reproducible compared to a single snapshot only, this method was chosen for the analysis in the present study.

		Summary statistics		Between-day variability	
Variable	Unit	Mean	s.d. (2.5-97.5th percentile)	CV (%)	RC
HR	Beats/min	33	2.3	6.8	6.27
EMAT	msec	96	14.3	14.9	39.655
EMATc	%	4.9	0.86	17.4	2.378
LVST	msec	508	15.8	3.1	43.658
LVSTc	%	28.2	1.38	4.9	3.822
EMAT/LVST	-	0.19	0.032	17.1	0.09
S3 (° max of 5)	Scale of 1-10	4.8	0.68	14.3	1.883
S4 (°max of 5)	Scale of 1-10	3.9	0.43	11.1	1.198
SDI	Scale of 1-10	2.2	0.34	15.6	0.931

Reproducibility of "snapshot" variables using the median° of 5 consecutive snapshots in 10 healthy horses. For a complete glossary of variables, see Table S3. CV, coefficient of variation; RC, repeatability coefficient according to the British standards institution; HR, heart rate; EMAT, electromechanical activation time; EMATc, rate-corrected electromechanical activation time; LVST, left ventricular systolic time; LVSTc, rate-corrected left ventricular systolic time; EMAT/LVST, ratio

of electromechanical activation time and left ventricular activation time; S3, power of the third heart sound; S4, power of the fourth heart sound; SDI, systolic dysfunction index.
